# Real-Time High Resolution THz Imaging with a Fiber-Coupled Photo Conductive Antenna and an Uncooled Microbolometer Camera

**DOI:** 10.3390/s21113757

**Published:** 2021-05-28

**Authors:** Peter Zolliker, Mostafa Shalaby, Elisa Söllinger, Elena Mavrona, Erwin Hack

**Affiliations:** 1 Transport at Nanoscale Interfaces Laboratory, Empa, Swiss Federal Laboratories for Materials Science and Technology, Überlandstrasse 129, 8600 Dübendorf, Switzerland; eleni.mavrona@empa.ch (E.M.); erwin.hack@empa.ch (E.H.); 2 Swiss Terahertz Research-Zurich, Swiss Terahertz GmbH, Technopark, 8005 Zurich, Switzerland and Park Innovaare, 5234 Villigen, Switzerland; shalaby@swissterahertz.com (M.S.); elisa.sollinger@swissterahertz.com (E.S.)

**Keywords:** THz imaging, real-time, photo conductive antenna, microbolometer camera, THz-TDS

## Abstract

We present a real-time THz imaging method using a commercial fiber-coupled photo conductive antenna as the THz source and an uncooled microbolometer camera for detection. This new combination of state-of-the-art components is very adaptable due to its compact and uncooled radiation source, whose fiber coupling allows for a flexible placement. Using a camera with high sensitivity renders real-time imaging possible. As a proof-of-concept, the beam shape of a THz Time Domain Spectrometer was measured. We demonstrate real time imaging at nine frames per second and show its potential for practical applications in transmission geometry covering both material science and security tasks. The results suggest that hidden items, complex structures and the moisture content of (biological) materials can be resolved. We discuss the limits of the current setup, possible improvements and potential (industrial) applications, and we outline the feasibility of imaging in reflection geometry or extending it to multi-spectral imaging using band pass filters.

## 1. Introduction

In material science, as well as in industrial and security applications, non-destructive testing of samples is an important prerequisite. Non-ionizing THz radiation can be an option, since it can deliver sub-millimeter resolution. Additionally, many materials have high transmissivity in this frequency range. A broad range of materials, such as plastics [1,2,3,4], ceramics [5,6], illicit drugs [7,8,9], explosives [8,9,10], wood [11,12,13], paper [14,15], leaves [10,16], and blood [17,18] have been successfully studied with THz radiation. A large number of security applications based on (sub-)THz radiation [8] have been proposed, and some are commercially available [8,19].

Despite the immense potential, the application of THz outside research is currently far from being common. In theory, a THz transmission imaging setup can be made out of a single line source, a collimating lens and a pixel array camera. This simplistic setup is a promising candidate for industrial and security applications. However, the achievable resolution and image quality, respectively, are limited by the irradiation wavelength, the numerical aperture (NA) of all optical components, as well as by the camera properties (pixel size, sensitivity etc.). To circumvent the limitations of the optical components, lens-less imaging [20,21] can be an option.

To date, the most commonly used sources in the frequency range between 0.2 and 4 THz are far infrared (FIR) gas lasers, quantum cascade lasers (QCLs) and photo-conductive antennas (PCAs).

The FIR gas lasers [22] are based on a high power, mid-infrared CO_2_-laser pumping a THz cavity. Their THz emission can be continuous-wave (cw), with the output power exceeding 150 mW at 2.52 THz [21]. The output wavelength depends on the gas in the THz resonator. However, cw lasers only emit a single line, and stable operation can be challenging. Recently, the relatively compact THz QCLs started performing without cryostat, operating with a thermoelectric cooler [23] and at temperatures up to 250 K [24]. In frequency combs operation, the bandwidth has been higher than an octave [25], but is still limited to 1–6 THz [26]. Recently, the reported peak output power reached 2 W (58 K, 3.3 THz, single mode) [27]. Despite the promising progress, more research is required to achieve room temperature operation, larger bandwidth and higher power.

PCAs combine many advantages of the above mentioned sources: They are compact, well established broadband sources with bandwidth up to 6 THz and a 90 dB dynamic range [28]. Their performance is limited by the near-infrared (NIR) pump pulse, carrier lifetime and the chosen detector. The majority of commercially available THz Time Domain Spectrometer (THz-TDS) use a PCA combined with off-axis parabolic mirrors (OAPMs) as a basis. Application of the compact and robust THz-TDS quickly spread from the first reported use case of water vapor absorption characterization [29] into other research disciplines, even including (art) conservation [30,31,32] and archaeology [6,32,33,34].

Thus far, for THz-TDS imaging, only prototypes of multipixel detectors [35] are reported; image acquisition requires a sequential scanning of the sample that cannot provide data in real-time. Nevertheless, scanning THz-TDS paved the way for the adaption of THz imaging in industrial applications, e.g., lacquer thickness determination [4,36]. As PCAs are widely available, THz imaging with them is very attractive. For example, Stantchev et al. used a PCA for real-time single pixel imaging [37].

Their method to modulate the THz beam via a digital micromirror device retains the time domain capabilities of the THz-TDS, whilst still achieving a resolution of 32×32 pixels at 6 frames-per-second (fps). Per contra, their approach requires elaborate equipment, whereas we propose a method based on a simple transmission setup, using a PCA as the source and exploiting the recent improvements of microbolometer cameras. Our approach can deliver much higher resolution and is more suitable for in-field (industrial) applications but sacrifices the spectral information.

In this paper, we give a short overview of the method, the camera properties, and the setups and describe the data processing. We recorded a THz beam shape in real-time and determined the spatial resolution with a Siemens star. The suitability of the method for practical applications was demonstrated by imaging a key concealed in a paper envelope, the qualitative resolution of different water content in leaves and the imaging of annual rings in wood. Finally, we discuss the limitations and possible improvements of the setup as well as suggest practical applications and future extensions.

## 2. Setups and Methods

### 2.1. Camera and Lens Properties

For the experiments, a RIGI camera and a THz lens (both Swiss Terahertz GmbH, Zurich, Switzerland) were used. Their specifications are found in Table 1 and Table 2, respectively.

The used camera, RIGI S2x, is a prototype that is optimized for low-frequency imaging. This is achieved through an optimized detector structure to enhance the absorption of low-frequency THz radiation.

### 2.2. Setups

A commercial THz-TDS system (Tera-FlashTF-1503, Version 4 December 2015, Toptica Photonics AG, Gräfelfing, Germany) was used as a starting point. In this system, a 100 μm InGaAs based strip-line antenna serves as the transmitter (TX). It is biased with 120 V and optically pumped by a pulsed 1550 nm Erbium fiber laser (pulse duration: 60 fs, repetition rate: 100 MHz). The 22.3 mW of NIR pump reaching the TX are converted to roughly 40 μW cw equivalent, linearly polarized THz radiation. The THz-TDS scan time was kept fixed at 70 ps throughout all the experiments.

**Figure 1:**
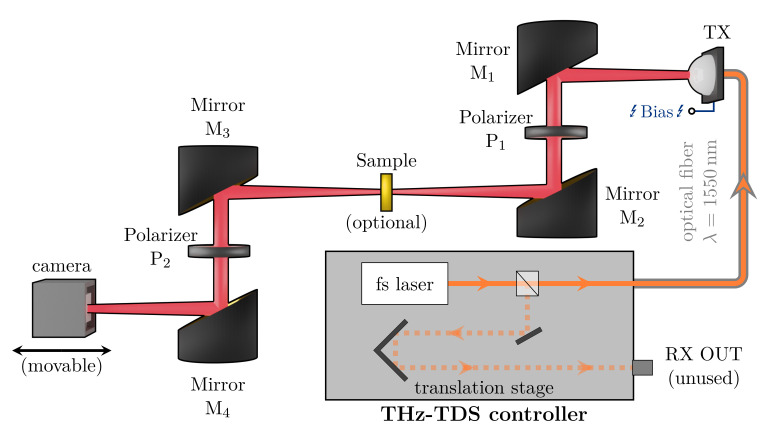
Schematic of the zigzag setup. Via an optical fiber, a fs pump laser (λ=1550 nm) excites the TX, which, in turn, emits THz radiation. Four OAPMs $M_{1}$ (f=89 mm), $M_{2}$ (f=178 mm), $M_{3}$ (f=178 mm) and $M_{4}$ (f=89 mm) and two polarizers, $P_{1}$ and $P_{2}$, guide the THz emission onto the camera sensor (positioned where in a THz-TDS the RX would be located).

The optical setup is of the zigzag-transmission geometry type (see Figure 1):

An OAPM collimates the diverging output of the TX, which is then focused by another OAPM. A sample can be placed in the beam waist. The transmitted radiation is guided by a second OAPM pair (rotationally symmetrical to the first one) onto the detector. In a standard THz-TDS, the detector would be a receiver (RX), working on the inverted principle of the TX. In this work, the RX was replaced with an uncooled microbolometer camera, which could be displaced along the THz propagation direction. Since the sensor is sensitive to all emitted wavelengths, the spectral resolution is not recoverable from the data.

**Figure 2:**
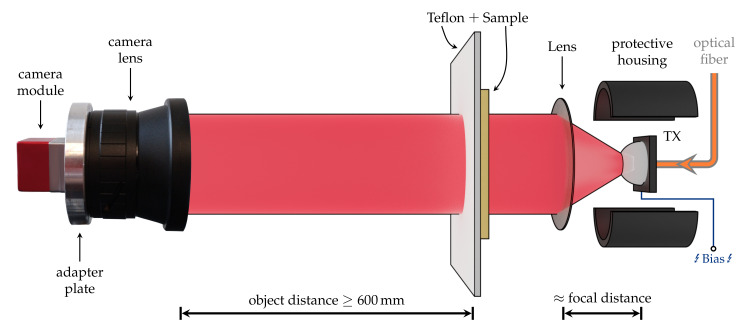
Schematic of the lens-based imaging setup. The THz emission of the TX is collimated by a silicon lens (f=25 mm) before it reaches the sample. To suppress the thermal image, the sample is mounted onto a sheet of Teflon. The transmitted radiation is recorded with the camera/lens combination (f=44 mm) placed more than 600 mm away from the object plane. Drawing not to scale.

On the other hand, a high real-time spatial resolution is achieved, in contrast to the single-pixel nature of the RX. Furthermore, two wire-grid polarizers were inserted in the parallel beam sections to ensure a high degree of linear polarization. Additionally, they also allow for intensity reduction via the rotating polarizer method. To simplify the setup of Figure 1, we removed all the OAPMs, illuminated the sample directly and captured the image with a lens specifically designed for the RIGI camera (Figure 2).

For practical imaging applications, the complicated OAPM alignment is less favorable than the simpler lens-based setup depicted in Figure 2. The TX is placed roughly in focus with a silicon (Si) lens (f=25 mm, d=32 mm), which collimates the divergent radiation of the THz emitter. The distance between the lens and PCA determines the size of the illuminated area. The majority of the samples were mounted close to the collimating lens on a 1 mm thick sheet of Teflon for thermal image suppression. If this was not possible, a 3 mm Teflon sheet was placed between the sample and the camera. Furthermore, black polyethylene (PE) foil was fixed to the TX to weaken the leaking 1550 mm NIR pump pulses. Due to the design of the camera lens (f=44 mm, fnumber=0.7), the minimum object distance was 600 mm.

### 2.3. Image Analysis and (Post-)Processing Routines

The pre-processed image data from the camera was sent via USB to a PC. A control software allowed for real-time filtering and saving of the data in different compressed and loss-less file formats. For this publication, the data were saved as 14 bit integers with minimal filtering into loss-less csv-files; only once compressed 8-bit jpg files were used.

In MATLAB, the following post-processing (also suitable for a real-time data stream) was applied: At first, dead pixels were removed by replacing them with a neighboring pixel. Optionally, images were then filtered with a 3×3 median filter followed by a Gaussian filter with a width of σ=1 pixel. Then, a background image, captured while the THz beam was blocked and pre-processed with the same procedure, was subtracted. Afterward, contrast enhancement was performed by re-scaling the minima/maxima of the gray-scale image data. For better visualization, some of the post-processed images were converted to false color.

Imaging of samples larger than the illuminated area was made possible by scanning over the sample in real-time and stitching the single frames together. The position offset between the neighboring frames was determined by using auto-correlation in an area around the center of the image.

The spatial scaling of the THz images was estimated from the sensor pixel pitch and known feature dimensions measured on the samples. Sample (feature) dimensions were extracted with ImageJ/Fiji (see e.g., [38,39]) from photographs of the samples on a graph paper background.

## 3. Results

### 3.1. THz-TDS Beam Profiling

**Figure 3:**
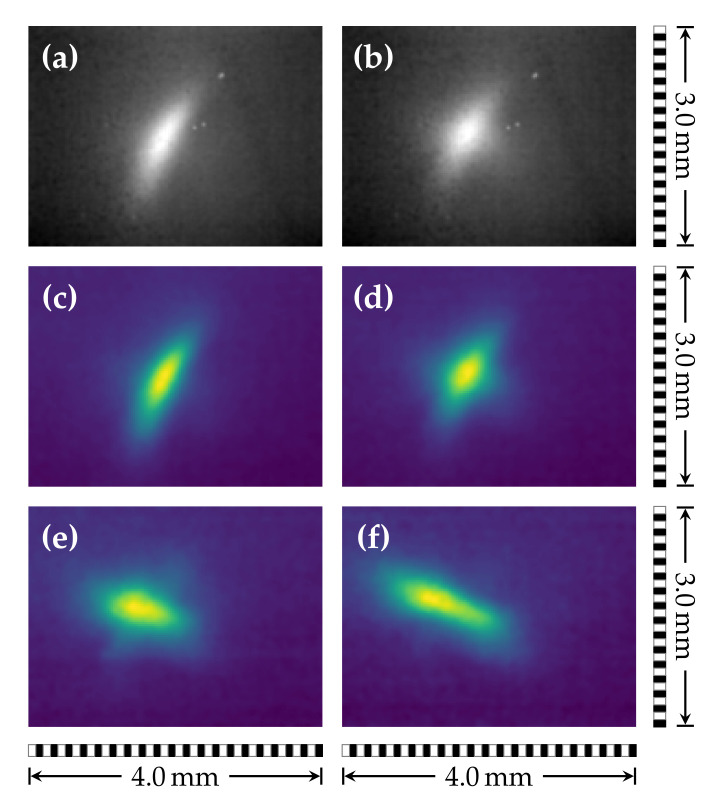
Selected single frames of a 1D real-time beam shape scan in the propagation direction. Data as saved from the camera software (**a**,**b**) and the same data for comparison in false-color with post-processing applied (**c**,**d**). Subfigures (**a**,**c**,**f**) depict the beam shape for two out-of-focus positions (before/after the focal point). The spatial intensity distribution close to the optimal focus is presented in (**b**,**d**,**e**).

As a first proof-of-concept, the beam profile of the PCA emission was measured with the setup depicted in Figure 1. In this configuration, we had a 1:1 imaging of the beam shape to the sensor. Since the intensity of the focused beam was too high for the extremely sensitive camera, the polarizer $P_{2}$ was rotated by $\theta \approx 65{}^{{}^{\circ}}$, letting, according to Malus’s law ($I=I_{0}\;\cdot \;\cos^{2}\left(\theta \right)$) [40], roughly 18% of the initial intensity pass. Assuming a 50% further loss along the optical path, we expected an average intensity of less than mW/m at the detector, but we were still able to obtain decent contrast without any data processing (see Figure 3a,b).

The images in Figure 3 represent single frames of a movie clip (see video S1 in Supplementary Materials ), which was obtained by moving the camera along the THz propagation direction. Data acquisition was performed at 9 fps, allowing the experimenter to receive immediate feedback. Even the unprocessed data directly streamed from the camera (Figure 3a,b) provided sufficient information for a qualitative analysis. Post-processing the camera data (Figure 3c–f) revealed that the beam out of focus (Figure 3c,f) was elliptical and tilted about $\pm 45{}^{{}^{\circ}}$ to the horizon. Close to the focus (Figure 3d,e), the beam was slightly cross-shaped. The continuous transition from $+45{}^{{}^{\circ}}$ to $-45{}^{{}^{\circ}}$ tilt could also be resolved.

### 3.2. Siemens Star

**Figure 4:**
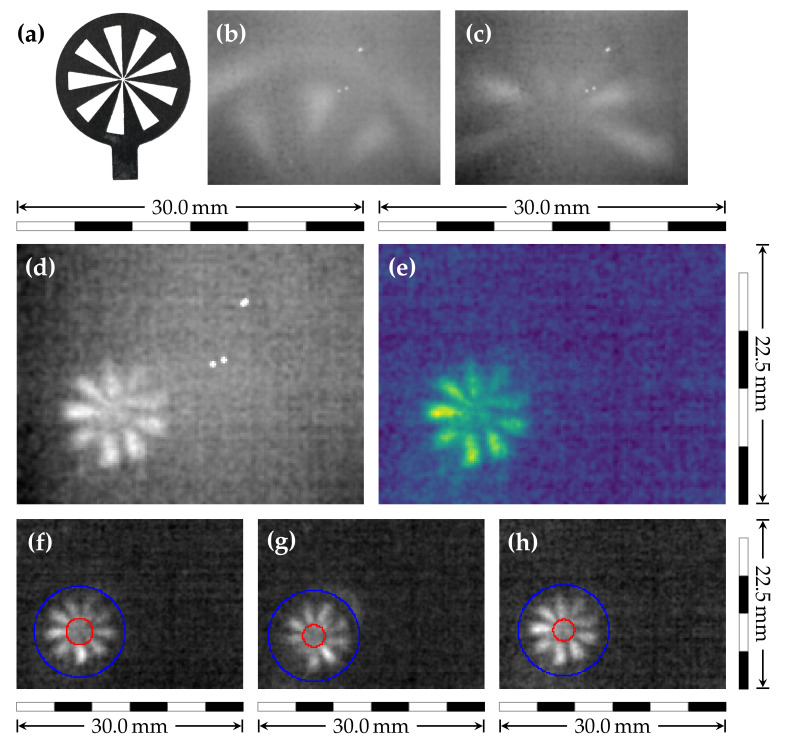
Imaging of a metallic Siemens star. Photography (VIS) of the Siemens star (**a**). Unprocessed THz images acquired in zigzag THz-TDS geometry (see Figure 1): Image with the outer rim of the Siemens star visible (**b**), whereas, in (**c**), only the central part is resolved. THz images acquired in lens-based setup (see Figure 2): unprocessed THz data (**d**) and false-color with dead-pixel-removal applied (**e**). Determination of the resolution shown with three example frames (**f**–**h**): The assumed outer rim of the Siemens star (blue circles) and resolution limit of 1.23 mm (**f**), 1.02 mm (**g**), and 0.99 mm (**h**) (red circles).

The first imaging tests were carried out on a Siemens star (photography with visible light (VIS) in Figure 4a, outer diameter d=12.5 mm, rim diameter $d_{\;\mathrm{rim}}=10.6\;mm$ and nine spokes), laser ablated from a thin metal sheet and mounted onto a 1 mm thick sheet of Teflon. To profit from the more intense sample irradiation in the THz-TDS (achievable intensity higher due to smaller beam size), the sample was placed into the standard position (see Figure 1). By intentionally shifting the first OAPM pair and TX closer to the sample, the focus is moved beyond the sample plane, effectively enlarging the illuminated sample area.

With this approach, a part of the Siemens star could be imaged (Figure 4b). However, the zigzag configuration with the OAPMs did not allow for an undistorted imaging of such a large sample. This was demonstrated by repositioning the Siemens star only slightly (Figure 4c). Switching to the linear setup (Figure 2) allowed to resolve the complete Siemens star (Figure 4d). For this data set, we did not use any spatial filtering to avoid its impact on the spatial resolution determination. Only dead pixel removal was applied (Figure 4e). The recorded real-time video nicely shows the rotation of the Siemens star (see Figure 4f–h and video S2 in Supplementary Materials) with some minor intensity fluctuations and shifts.

The quality of these images allow an estimation of the spatial resolution. First, the smallest radius $r_{\min}$ of a centered circle is determined for which the average contrast between a spoke and an opening is larger than 10% of the highest contrast. Then, the resolution is $r_{\mathrm{res}}=2\pi \;\cdot \;r_{\min}/N$, where N=9 is the number of spokes. For the current imaging setup, a resolution $r_{\mathrm{res}}=1.05\left(15\right)mm$ was estimated from ten different Siemens star images.

### 3.3. Key in an Envelope

**Figure 5:**
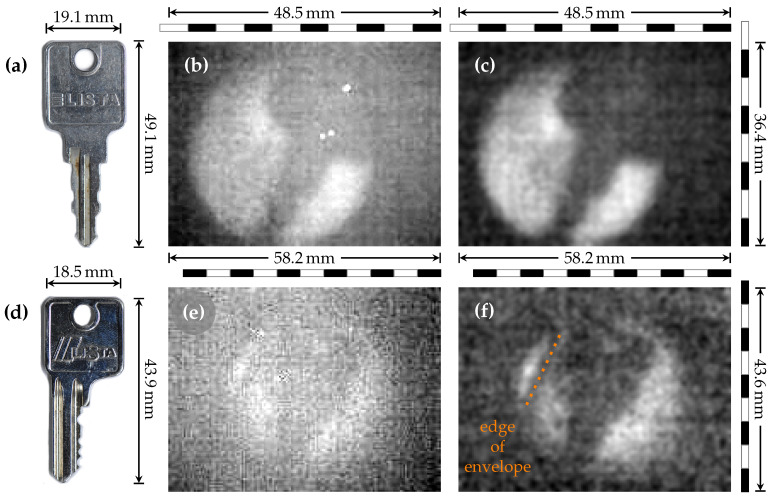
Imaging of the metal keys. Photography of the keys with rough dimensions (**a**,**d**), raw THz images (**b**,**e**) of the keys, and post-processed THz images (**c**,**f**) of the keys with dead pixel removal. A key can still be resolved within a standard paper envelope (**e**,**f**), with the edge of envelope marked (**f**).

We demonstrate the capability of our method for detecting concealed (metallic) objects from a larger distance by examining two metallic keys, (Figure 5a,d), one of them concealed with a standard paper envelope. Each of them was placed into the THz beam approximately 600 mm away from the camera assembly (see Figure 2). The thermal image was suppressed with 3 mm of Teflon, located between the sample and camera. As expected, the metal key without envelope was clearly resolved (Figure 5b). The post-processing enhanced the contrast and made the edges more defined (Figure 5c, video S3 in the Supplementary Materials). For the concealed key, the image quality was reduced due to the absorption and diffraction on the rough paper surface (Figure 5e).

Additional quality loss originated from saving the data for testing purposes as 8-bit jpg files, a format that appears to not be suitable for our THz imaging purposes. Overall, the key shape was fairly faint, but post-processing was able to enhance the visual clarity so that even the edge of the paper envelope became visible (Figure 5f). The video S4 in the Supplementary Materials shows how the experiment was performed in the laboratory.

### 3.4. Leaves with Different Moisture Contents

**Figure 6:**
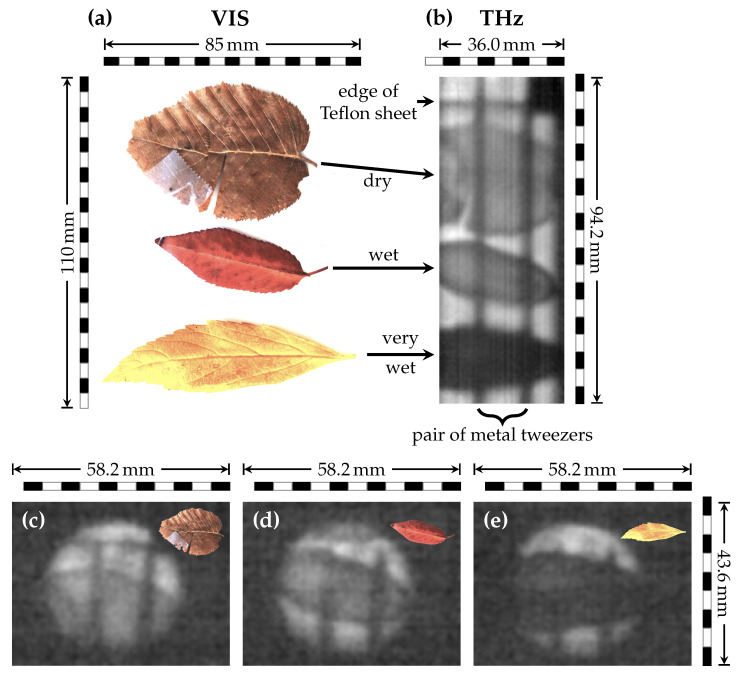
(**a**) Photography (VIS) of leaves with different water contents. One leaf stored in a drawer for five years (top) and two found just prior to the experiments: one from a dry place (middle) and one from a wet gutter (bottom). Glued with THz transparent adhesive tape onto a 1 mm thick Teflon sheet, Teflon and tape stripes removed by image processing for better visual clarity, and partially still visible. THz image of the different leaves (**b**) with a pair of tweezers for contrast enhancement (vertical lines) and the edge of the Teflon sheet (top, horizontal) visible. Higher water contents are clearly represented by decreased brightness. Image stitched with auto-correlation from single frames of a real-time 1D scan. An exemplary post-processed single frame is presented for the dry (**c**), wet (**d**) and very wet (**e**) leaves.

The strong absorption of water in the THz regime renders THz imaging an interesting modality for biological samples. We evaluated the potential of our approach by investigating leaves with different moisture contents. Three distinct leaf specimens (Figure 6a) were mounted on a 1 mm thick Teflon sheet and were scanned as described in Section 2.3 and video S6 in the Supplementary Materials. The stitched image (Figure 6b) as well as exemplary single frames (Figure 6c–e from video S5 in the Supplementary Materials) provide the same distinct larger features, including the shape, cracks etc., as shown in the photography (Figure 6a). Additionally, the THz image showed leaves with higher moisture content as distinctly darker. Despite losing the ability to resolve finer details, this could allow for an accurate qualitative analysis and even monitoring of diffusion processes in real-time.

### 3.5. Thin Wood Sample

A 0.19 mm thin, microtome-cut wood sample was mounted in a rotatable holder. Thermal image suppression was achieved with 3 mm of Teflon, located between sample and camera. The zero position of the rotation ($\varphi =0{}^{{}^{\circ}}$) was defined such that the annual rings were parallel to the polarization of the THz radiation. This preventive measure allowed us to determine whether any influence of the polarization on the recorded image existed.

**Figure 7:**
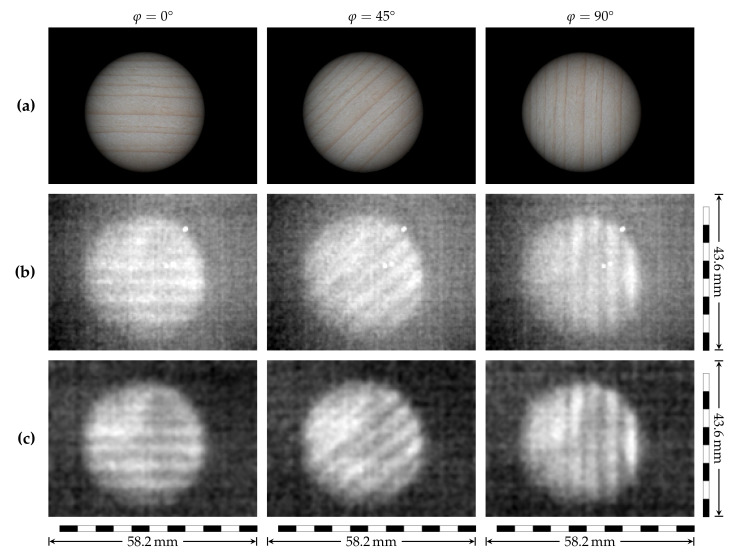
Imaging of a thin wood sample. (**a**) Not to scale artistic illustration of the approximate illumination of the thin wood sample for different angles φ, corresponding to the selected THz single frames from a full sample rotation recorded in real-time (**b**,**c**). Unprocessed data (**b**) as obtained from the camera and post-processed representation with dead pixel removal (**c**).

An approximation for the illuminated area of the actual sample is shown for different orientations in the artistic illustration Figure 7a. The annual rings are already visible in the raw THz images (Figure 7b) and become more pronounced in the post-processed data (Figure 7c). The annual rings are clearly recognizable for each configuration. There is no evidence that the ring orientation would influence the image contrast. The THz images (Figure 7b,c) are single frames of a video clip (video S7 in Supplementary Materials) that we were able to record in real-time despite the high absorption.

## 4. Discussion

### 4.1. Limitations and Potential Improvements of the Current Setup

The major limitation of using a PCA as a THz source is the low output power. This is less relevant as long as images are acquired with a focused beam. However, for a collimated beam, the irradiance decreases quadratically with the beam radius. For example, a strong transmission signal through plastic (food) containers could be detected in the THz-TDS (focused, RX); however, with the collimated beam and camera instead of RX, no valid signal could be recorded.

Although the camera is extremely sensitive, the expanded beam combined with the sample absorption did not provide enough irradiance at the sensor for real-time imaging. For the current imaging setup, a resolution $r_{\mathrm{res}}=1.05\left(15\right)mm$ was estimated from the Siemens star images (Figure 4c–f). This resolution is not far from the maximum that we can expect if we compare it with the full width at half maximum of the smallest beam shape (Figure 3d,e) of 0.65(10)mm and the wavelength of maximal intensity of the THz-TDS (0.6 mm corresponding to 0.5 THz), which determines the maximal achievable resolution.

We presume that one relevant contribution to the limited resolution is the use of a broadband emitter instead of a single line source. Although the radiation contains high frequencies, which would allow for better spatial resolution, the dominant signal from lower frequencies blurs the image and dominates the resolution properties. Furthermore, the strong water vapor absorption under ambient conditions greatly reduces the intensity of shorter wavelengths, thus, leaving only longer wavelengths available for imaging.

Since the image quality highly depends upon the optical path length, reducing the overall distance between the source, sample and camera in conjunction with suppressing the water vapor influence would help. If working over larger distances or under ambient conditions is absolutely required, the ongoing development of more efficient PCAs could provide a larger frequency bandwidth and higher intensities at shorter wavelengths. Blocking the lower frequencies with a high pass filter whilst still maintaining enough intensity for a high contrast THz image would allow for resolving smaller structures that are not accessible with the current setup.

### 4.2. Potential Applications

For the THz community, real-time beam profiling of weak and/or broad band sources like shown in Figure 3 is still a difficult task. The results shown in this work also suggest that the new generation of very sensitive THz microbolometer cameras can image beams emitted from a PCA as well as other weak THz sources. The sample thickness is a relevant factor in potential imaging applications. The main limitations are the absorption properties of the sample at 0.5–1 THz. In particular for samples containing water, the sample thickness is limited to the sub mm range. The focusing properties of the beam have to be considered if the samples are thicker than the wavelength.

With the proof-of-concept that complex sample structures can be imaged (Figure 6 and Figure 7), the setup described in this work can also provide material scientists with easier access to THz experiments. These types of experiments include polymers, aerogels, (embedded) nanomaterials and materials derived from modified biological precursors. The latter class can also act as a bridge to biology. In-field, in vivo experiments on leaves, grass, crops and young (tree) saplings are all within the realms of possibility. Due to the comparatively high portability and low power consumption and robustness, long term field experiments in remote locations without infrastructure/off-grid seem feasible.

From the capability to image biological samples, the agriculture and food industry could also profit directly, e.g., improving water management by monitoring the water content of leaves and plants. This has been demonstrated before with various THz setups [16]. This imaging method could not only be applied during the production but could also ensure the product quality during transport and further processing, e.g., the real-time detection of spoiled products or foreign bodies through the packaging (see Figure 5). The latter also immediately implies suitability for security applications, such as mail screening.

Further industrial use cases could include quality control during production, e.g., the monitoring of the water content in paper and the safety inspection or recycling of plastics. THz photoelasticity, where one measures the stress states of THz-transparent materials in transmission, would also be conceivable. The broad spectral range and polarization control can be used to visualize and evaluate residual stress distributions in packaging materials and electronic housings as well as to display stress distributions in real-time during a mechanical test.

### 4.3. Outlook: Possible Modifications

Since imaging in the THz-TDS zigzag geometry has been shown (Figure 4b,c), imaging using a PCA is not restricted to a conventional lens-based THz microscope. Instead of using lenses, setups with OAPMs specially designed for imaging are possible. This could allow accessing methods/samples that cannot be used/measured with commercially available (macro) lenses. A major disadvantage for achieving good image quality with this concept is the many degrees of freedom of an OAPM. This makes precise alignment very challenging; however, exact calibration is an essential prerequisite for obtaining a high quality image.

During our initial experiments with the new source-detector combination, we focused on transmission. However, it is straightforward to change the setup from transmission to reflection imaging by rearranging the illumination geometry. To mitigate the loss of the time domain/spectral information caused by using a microbolometer camera instead of a conventional RX, we propose multi-spectral imaging via inserting THz band pass filters into the beam path. Mounting the filters onto a rotating wheel could allow the generation of false-color images in real-time. Its feasibility depends upon the quality of the THz filters and the availability of PCA sources with high power.

## 5. Conclusions

We presented a THz real-time imaging method based upon a fiber-coupled PCA and an uncooled microbolometer camera with 120 × 160 pixels. As a proof-of-concept, we recorded a THz-TDS beam shape in real time at nine frames per second and examined the performance for typical security, quality control and material science tasks in transmission geometry. The challenges encountered during the experiment were the weak sample irradiance, a resolution lower than the physical wavelength limit and, for some samples, reduced image quality. We discussed possible improvements and (practical) applications of the setup, including experiments in reflection geometry and multi-spectral imaging with THz filters.

## Figures and Tables

**Table 1:** Technical specification of the camera.

Camera	Swiss Terahertz RIGI S2x
Type	uncooled THz microbolometer
Operation range	16–3000 μm(0.1–18 THz)
Pixel size (μm)	25
Number of pixels	160×120
Detector size (L × H, ${mm}^{2}$)	4×3
NEP	<$1.5pW/\sqrt{\mathrm{Hz}}$ @ 4.6 THz
ADC (bit)	14
Frame transfer rate (fps)	9
Data transfer + power	USB

**Table 2:** Technical specification of the lens.

Lens	Swiss Terahertz Objective Lens
Focal length (mm)	44
f number	0.7
Lens material	HRFZ-Si
FoV (lateral×vertical)	$17.3{}^{{}^{\circ}}\times 13{}^{{}^{\circ}}$
Operation range	7.4–3000 μm(0.1–30 THz)

## Data Availability

The data presented in this study are available on request from the corresponding author.

## Supplementary Materials

The following are available online at https://www.mdpi.com/article/10.3390/s21113757/s1, Video S1: Real-time THz-TDS beam profiling, Video S2: Real-time rotation of a metallic Siemens star, Video S3: Real-time THz imaging of a key, Video S4: Demonstration of laboratory equipment with a concealed key, Video S5: Real-time moisture content resolution in leaves, Video S6: Demonstration of laboratory equipment with wet leaves, and Video S7: Real-time rotation of a thin wood sample.

## Abbreviations

The following abbreviations are used in this manuscript (alphabetical order):

ADC	Analog to Digital Converter
cw	Continuous wave
FIR	Far Infrared
FoV	Field Of View
fps	Frames per second
fs	Femto second
HRFZ-Si	High Resistivity Float Zone Silicon
NA	Numerical Aperture
NEP	Noise Equivalent Power
NIR	Near-infrared
OAPM	Off-Axis Parabolic Mirror
PCA	Photo Conductive Antenna
PE	Polyethylene
ps	Pico second
QCL	Quantum Cascade Laser
RX	Receiver
Si	Silicon
TDS	Time Domain Spectroscopy/Spectrometer
THz	Terahertz
TX	Transmitter
VIS	Visible spectrum
